# The RING-domain E3 ubiquitin ligase OsRGLG6 regulates rice grain number and yield via ubiquitination-mediated degradation of OsOTUB1

**DOI:** 10.1007/s42994-025-00232-5

**Published:** 2025-07-23

**Authors:** Jia Chen, Huixia Song, Chenyang Xu, Pengfei Wang, Shuansuo Wang

**Affiliations:** 1 Shanxi Houji Laboratory, Shanxi Agricultural University, Taiyuan, 030031 China; 2 Journal Center, Shanxi Agricultural University, Taiyuan, 030031 China

**Keywords:** Rice yield, Grain number per panicle (GPP), OsOTUB1, OsRGLG6, E3 ubiquitin ligase, Ubiquitination

## Abstract

Rice (*Oryza sativa*) yield is a complex trait determined by multiple factors, including grain number per panicle (GPP), which is mainly genetically regulated. In this study, we identified the E3 RING DOMAIN LIGASE (RGLG) OsRGLG6 as a key regulator of GPP in rice. A yeast two-hybrid screen revealed that OsRGLG6 interacts with OsOTUB1, an ovarian tumor domain-containing ubiquitin-specific protease involved in panicle development. Further experiments confirmed the interaction between OsRGLG6 and OsOTUB1 in vitro and in vivo. OsRGLG6, which contains a vWA domain and a RING domain, is localized in the cytoplasm and endoplasmic reticulum and is highly expressed specifically in rice panicles. In vitro and in vivo ubiquitination assays demonstrated that OsRGLG6 ubiquitinates OsOTUB1 and promotes its degradation via the 26S proteasome pathway. CRISPR/Cas9-generated *osrglg6* mutants exhibited significant reductions in GPP, indicating a crucial role for OsRGLG6 in the regulation of grain number. Transcriptome analysis revealed that OsRGLG6 regulates multiple downstream genes involved in plant hormone signaling, nitrogen utilization, and stress response, and the *OsRGLG6*–*OsOTUB1* module specifically mediated drought-stress responses in a simulated drought experiment. This study provides insight into the molecular mechanisms that regulate rice GPP and yield, highlighting the potential of *OsRGLG6* as a target for improvement of rice productivity.

**Supplementary Information:**

The online version contains supplementary material available at 10.1007/s42994-025-00232-5.

## Introduction

Rice (*Oryza sativa*), a staple food for a substantial proportion of the global population, has long been the focus of intensive research efforts aimed at improving its yield. Rice yield, defined as the total grain output per unit area, is a complex trait predominantly determined by three factors: tiller number, grain number per panicle (GPP), and thousand-grain weight (Lu et al. 2022; Xing and Zhang 2010). GPP is regarded as one of the most critical yield determinants (Gouda et al. 2020; Huo et al. 2017; Wu et al. 2021). It serves as a direct barometer of plant potential for grain production, and fluctuations in GPP profoundly influence overall yield.

GPP is determined by a complex interplay of genetic factors, and previous investigations have identified several genes and genetic pathways that modulate grain number. For instance, *MONOCULM 1* (*MOC1*) has fundamental roles in the regulation of axillary meristems and subsequent bud outgrowth, as well as the control of tiller and panicle morphogenesis. Mutations in *MOC1* can lead to aberrant panicle architectures, significantly altering the number of grains per panicle (Li et al. 2003; Zhang et al. 2021). Allelic variations in *DENSE AND ERECT PANICLE 1* (*DEP1*), which encodes the C-type Gγ subunit, have been associated with changes in panicle density and grain number. The dominant allele *dep1* at this locus represents a gain-of-function mutation and produces phenotypes such as shorter, more upright panicles, increased grain density, and more grains per panicle (Huang et al. 2009, 2022). *SQUAMOSA PROMOTER BINDING PROTEIN-LIKE 14* (*OsSPL14*) encodes a transcription factor that regulates panicle branching as part of a complex genetic network. It not only is negatively regulated by OsmiR156 but also binds to the promoters of *TEOSINTE BRANCHED 1* (*OsTB1*), a negative regulator of rice tillering, and *DEP1* (Jiao et al. 2010; Lu et al. 2013)*.* Both overexpression and knockdown of *OsSPL14* can significantly affect panicle branching, increasing or reducing it and thereby affecting grain-production potential (Jiao et al. 2010; Miura et al. 2010). *OsOTUB1* encodes an ovarian tumor domain-containing ubiquitin-specific protease, and its reduced expression promotes meristem activity, decreases tiller number, and increases grain number per panicle and grain weight, ultimately improving rice yield. Although the precise molecular mechanisms of OsOTUB1 function remain to be characterized, it is known to participate in the regulation of SPL14 stability through K63-linked ubiquitination (Huang et al. 2017; Wang et al. 2017).

In addition to panicle architecture, the timing of flowering also has a significant influence on panicle development and, ultimately, GPP (Lu et al. 2022). *HEADING DATE 1* (*Hd1*) encodes an ortholog of *CONSTANS* from* Arabidopsis thaliana* (Arabidopsis) that functions primarily to promote flowering under short-day conditions and repress flowering under long-day conditions (Endo-Higashi et al. 2011; Yano et al. 2000). *HEADING DATE 3a* (*Hd3a*) is also involved in the flowering process and is regulated by *Hd1* (Itoh et al. 2010; Kojima et al. 2002). *GRAIN NUMBER*, *PLANT HEIGHT*, *AND HEADING DATE 7* (*Ghd7*) encodes a CCT-motif protein that represses flowering regardless of day length, and *DAYS TO HEADING 8* (*DTH8*) also influences the onset of flowering (Wei et al. 2010; Zong et al. 2021). The combination functional of *Hd1* and *EARLY HEADING DATE 1* (*Ehd1*) can reduce the number of primary branches per panicle, resulting in fewer spikelets per panicle (Endo-Higashi et al. 2011). Delayed flowering, often associated with an increase in grain number, affords the plant additional time for the development of supplementary branches and florets and thereby enhancing the potential for higher GPP. Enhanced expression of *Ghd7* under long-day conditions delayed heading and increased plant height and panicle size (Xue et al. 2008). *DTH8* delayed flowering under long-day conditions and increased the number of tillers and primary and secondary branches, resulting in 50% more grains per plant (Yan et al. 2011).

Plant hormones, particularly cytokinin, also play pivotal parts in regulating panicle number and thus GPP. Genes encoding the cytokinin-activating enzyme LONELY GUY (LOG) and CYTOKININ OXIDASE 2 (CKX2) both strongly affect the number of grains per panicle in rice (Ashikari et al. 2005; Kurakawa et al. 2007; Sandhu et al. 2024). LOG promotes the activation of cytokinin, which is required for panicle development, whereas CKX2 degrades cytokinin, thereby fine-tuning cytokinin homeostasis (Kurakawa et al. 2007). *ERECTA1* (*OsER1*) modulates the OsMKKK10–OsMKK4–OsMPK6 signaling pathway, which in turn regulates gibberellin metabolism, ultimately influencing grain number (Guo et al. 2020b). The gibberellin synthase gene *GA20ox1* and the auxin synthesis gene *TILLERING AND SMALL GRAIN 1* (*TSG1*) are also involved in GPP regulation. *GA20ox1* transcript levels were higher in the near-isogenic line NIL-*GNP1*^TQ^ than in its isogenic control and were associated with significantly higher grain number and yield (Wu et al. 2016a, b). The *tsg1* mutant exhibited increased sensitivity to indole acetic acid and L-kynurenine, an auxin biosynthesis inhibitor that targets tryptophan aminotransferases. Knockout of *TSG1* increased tiller number but reduced grain number, grain size, and plant height (Guo et al. 2020a).

Ubiquitination, a post-translational protein modification, is involved in diverse cellular processes, including protein degradation, modulation of protein trafficking, and regulation of various signaling pathways. This process is executed through a three-step enzyme cascade involving E1 (ubiquitin-activating enzyme), E2 (ubiquitin-conjugating enzyme), and E3 (ubiquitin ligase) (Copeland and Li 2019; Pickart 2001; Sharma et al. 2016). E3 ligases have attracted particular attention owing to their high specificity in targeting proteins for ubiquitination. There are four principal types of E3 ligase: Homologous to the E6-AP Carboxyl Terminus (HECT), Really Interesting New Gene (RING), U-box, and Cullin-RING (CRL) (Copeland et al. 2019; Zheng and Shabek 2017; Vierstra 2009). RING DOMAIN LIGASEs (RGLGs), which belong to the RING-type ligase family, have a von Willebrand factor type A (vWA) structural domain at their N terminus and a RING structural domain at their C terminus (Zhang et al. 2024; Wang et al. 2020). The five RGLG proteins in Arabidopsis participate in the regulation of various biological processes. RGLG1 and RGLG2 negatively modulate MAPKKK18-mediated drought-stress tolerance by ubiquitinating MAPKKK18 and promoting its degradation (Yu et al. 2021). In addition, they interact with AtERF53 and mediate its ubiquitination for proteasomal degradation, negatively regulating the drought-stress response (Cheng et al. 2012). RGLG2 also catalyzes the formation of ubiquitin Lys-63 chains and participates in auxin-regulated apical dominance (Yin et al. 2007). RGLG3 and RGLG4 are essential for jasmonate-mediated responses: they modulate JA-inhibited root growth, response to coronatine, and susceptibility to pathogens, as well as the JA-dependent wound response (Nagels Durand et al. 2016; Zhang et al. 2012). Moreover, the ubiquitin-conjugating enzyme UBC13, which can form Lys-63-linked ubiquitin chains, is required for formation of branched root hairs in the context of the iron-deficiency response (Pan et al. 2014). RGLG1 and RGLG2 can interact with UBC13 and are involved in related processes (Yin et al. 2007). OsRGLG5 recognizes the *Magnaporthe oryzae* effector AvrPi9 and promotes its ubiquitination, thereby positively regulating basal resistance to rice blast (Liu et al. 2023).

OsOTUB1 is a key regulator of grain number in rice and also modulates plant architecture. OsOTUB1 was previously reported to be ubiquitinated in young rice panicles (Zhu et al. 2020), but the specific factors that control its stability are not fully understood. In this study, we identified the E3 ubiquitin ligase OsRGLG6 through a yeast two-hybrid library screen and demonstrated its ability to modulate the stability of OsOTUB1. In vitro and in vivo ubiquitination assays unequivocally demonstrated that OsRGLG6 can ubiquitinate OsOTUB1 and facilitate its degradation via the 26S proteasome pathway. Our findings offer insight into the molecular mechanisms that regulate rice grain number and yield and may contribute to the development of strategies for enhancing rice productivity.

## Results

### The E3 ubiquitin ligase OsRGLG6 interacts with OsOTUB1

**Fig. 1:**
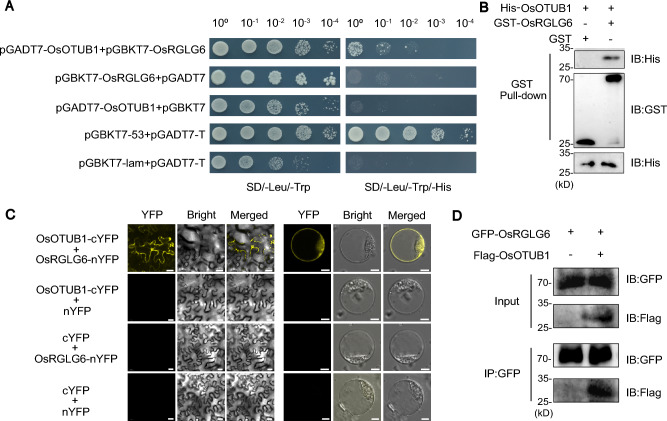
OsRGLG6 interacts with OsOTUB1. **A** Yeast two-hybrid assay demonstrating the interaction between OsOTUB1 and OsRGLG6. The yeast cells were grown on SD/–Leu/–Trp (left) and SD/–Leu/–Trp/–His (right) media. **B** In vitro pull-down assay showing the interaction between OsOTUB1 and OsRGLG6.** C** BiFC assay showing the interaction between OsRGLG6 and OsOTUB1 in *N. benthamiana* (left, scale bars = 30 μm) and rice protoplasts (right, scale bars = 10 μm). Each BiFC assay was performed at least three times. **D** Co-immunoprecipitation assay in *N. benthamiana*. Proteins were detected using anti-Flag (Flag-OsOTUB1) and anti-GFP (GFP-OsRGLG6) antibodies

Because full-length OsOTUB1 is toxic and inhibits yeast growth, we used the C terminus (198 aa) of OsOTUB1 for a yeast two-hybrid (Y2H) library screen to identify its interacting proteins (Fig. S1). We identified an intriguing E3 ubiquitin ligase, OsRGLG6, that interacted with the C terminus of OsOTUB1 (Table S1, Fig. 1A). We performed GST pull-down assays to verify this interaction in vitro and found that the purified GST-OsRGLG6 fusion protein specifically interacted with His-OsOTUB1, whereas GST alone did not (Fig. 1B). To confirm this interaction in vivo, we performed a bimolecular fluorescence complementation (BiFC) assay in *Nicotiana benthamiana*. Co-expression of OsRGLG6-nYFP and OsOTUB1-cYFP in *N. benthamiana* epidermal cells resulted in strong YFP fluorescence signals predominantly in the cytoplasm, with weaker signals in the nucleus (Fig. 1C). This result was confirmed by a BiFC assay in rice protoplasts (Fig. 1C), and a co-immunoprecipitation (Co-IP) assay also revealed that OsRGLG6-GFP associated with OsOTUB1-Flag (Fig. 1D). Collectively, these results provide robust evidence for the interaction between OsRGLG6 and OsOTUB1, both in vitro and in vivo.

### OsRGLG6 reduces OsOTUB1 stability without affecting its subcellular distribution

OsRGLG6, which belongs to the RING-type E3 ubiquitin ligase family, contains two distinct domains: a conserved vWA domain and a RING finger domain (Fig. S2A). The vWA domain mediates protein–protein interactions, and the RING domain confers E3 ubiquitin ligase activity and facilitates the transfer of ubiquitin from E2 enzymes to substrate proteins (Zhang et al. 2024). Because the RGLG gene family has multiple members in Arabidopsis and rice, we constructed a phylogenetic tree using the five RGLGs from Arabidopsis and the six most similar genes from rice, including OsRGLG6 (Fig. S2B). OsRGLG6 was most closely phylogenetically related to OsRGLG5 in rice and to AtRGLG5, AtRGLG1, and AtRGLG2 in Arabidopsis. Conserved-motif analysis identified eight motifs in OsRGLG6 (motifs 1–7 and 9), and motifs 1, 2, 3, 5, and 9 were present in all rice RGLG family members (Fig. S2C). On the basis of its identification as a RING-type E3 ubiquitin ligase, we proposed that OsRGLG6 might regulate OsOTUB1 protein stability.

**Fig. 2:**
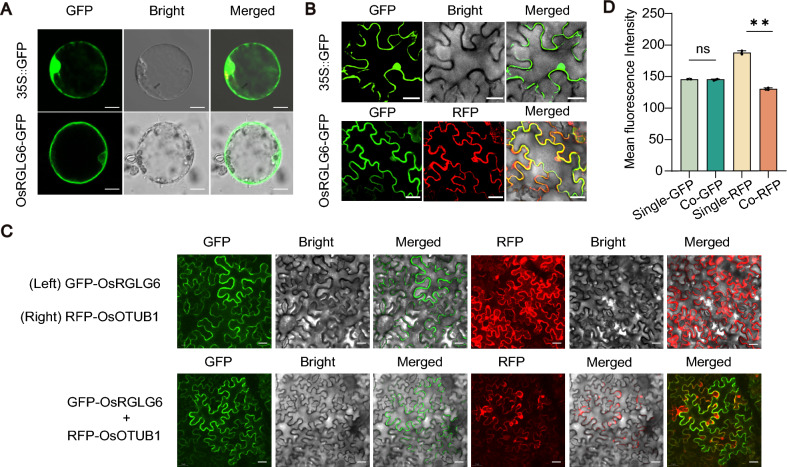
Subcellular localization of OsRGLG6 and its destabilization of OsOTUB1. **A, B** Subcellular localization of OsRGLG6-GFP in rice protoplasts (**A**; scale bars = 10 μm) and *N. benthamiana* epidermal cells (**B**; scale bars = 30 μm). All experiments were performed three times under identical conditions. **C** Subcellular localization analysis showed that OsRGLG6 mediated the degradation of OsOTUB1 without altering OsOTUB1 localization. Co-expression of GFP-OsRGLG6 and RFP-OsOTUB1 significantly reduced the fluorescence intensity of RFP-OsOTUB1. Scale bars = 30 μm. **D** Mean fluorescence intensity. Values are means ± SEM (*n* = 3). **P < 0.01; ns, no significant difference (one-way analysis of variance (ANOVA))

Because the spatial distribution of E3 ligases often determines their access to substrate proteins, we next investigated the subcellular localization of OsRGLG6 using rice protoplasts. OsRGLG6-GFP fluorescence was observed mainly in the cytoplasm and at the outer periphery of the nucleus, with a minor presence inside the nucleus, whereas GFP alone was localized diffusely in the cytoplasm and nucleus (Fig. 2A). This pattern of OsRGLG6 localization was consistent with previous reports for OsRGLG4 and OsRGLG5 (Choi and Jang 2023; Lim et al. 2013; Liu et al. 2023). An online prediction tool (WoLF PSORT) suggested that OsRGLG6 was also localized to the endoplasmic reticulum (ER). We tested this prediction by co-expressing OsRGLG6-GFP with an ER marker in *N. benthamiana* and observed partial overlap between the fluorescence signals of OsRGLG6-GFP and the ER marker (Fig. 2B). The cytoplasmic and ER localization of OsRGLG6 suggests that it may encounter and regulate cytoplasmic pools of OsOTUB1. We, therefore, co-expressed GFP-OsRGLG6 and RFP-OsOTUB1 in *N. benthamiana* leaves and found that OsRGLG6 did not alter the localization of OsOTUB1 but caused a significant reduction in OsOTUB1 protein levels, suggesting that OsRGLG6 promotes the degradation of OsOTUB1 (Fig. 2C, D).

### Loss of *OsRGLG6* function significantly reduced grain number per panicle

To clarify the spatiotemporal expression patterns of *OsRGLG6*, we examined its expression in roots, shoots, stems, leaves, leaf sheaths, 0.2 cm panicles, and 0.5-cm panicles. OsRGLG6 was highly expressed in 0.5 cm panicles and roots, but its expression was relatively low in leaves and shoots (Fig. S3). These findings are consistent with previous research and with the expression-prediction data available from the Rice Expression Database (https://ngdc.cncb.ac.cn/red/index), suggesting that OsRGLG6 plays a significant role in panicle development.

**Fig. 3:**
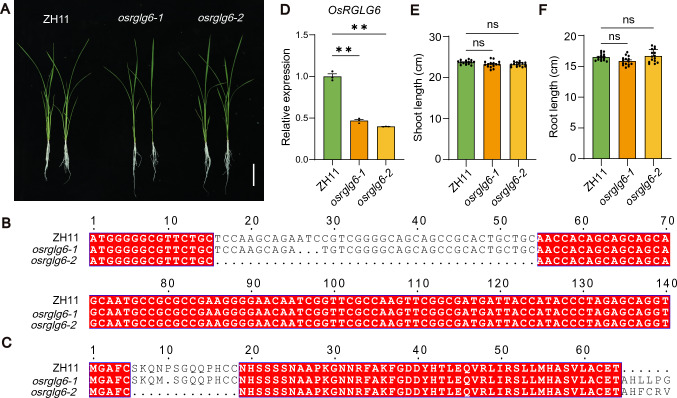
Seedling phenotypes and molecular characterization of the *osrglg6* mutants. **A** Seedling phenotypes of ZH11, *osrglg6-1*, and *osrglg6-2*. Scale bar = 5 cm. **B** DNA sequence alignment of target regions in ZH11, *osrglg6-1*, and *osrglg6-2*. **C** Protein sequence alignment of the corresponding regions in ZH11, *osrglg6-1*, and *osrglg6-2.* **D** Relative expression levels of *OsRGLG6* in ZH11, *osrglg6-1*, and *osrglg6-2*. Values are means ± SEM (*n* = 3). **P < 0.01 (one-way analysis of variance (ANOVA) comparing mutants to ZH11). Shoot length **E** and root length **F** of ZH11, *osrglg6-1*, and *osrglg6-2*. Values are means ± SD (*n* = 15). Data were analyzed by one-way analysis of variance (ANOVA), comparing mutants to ZH11; ns indicates that there was no significant difference

To investigate the role of *OsRGLG6* in grain-number regulation, we created CRISPR/Cas9 loss-of-function mutants (*osrglg6-1* and *osrglg6-2*) in the ZH11 background (Fig. 3A). Sequencing revealed that *osrglg6-1* had a 3-bp deletion that led to the substitution of asparagine with methionine and the deletion of proline, and *osrglg6-2* had a 39-bp deletion that resulted in the loss of 13 amino acids (Fig. 3B, C). Reverse transcription quantitative PCR (RT-qPCR) confirmed that *OsRGLG6* transcript levels were reduced in both mutants compared with wild-type ZH11 (Fig. 3D).

**Fig. 4:**
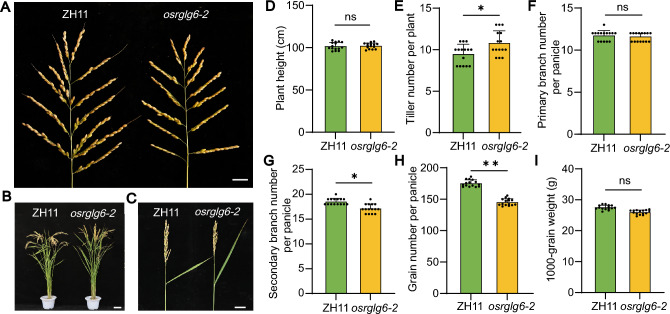
Phenotypic and agronomic characterization of *osrglg6-2*. **A** Panicle morphology of ZH11 and *osrglg6-2*. Scale bar = 2 cm. **B** Mature plant morphology of ZH11 and *osrglg6-2*. Scale bar = 10 cm. **C** Leaf angle of ZH11 and *osrglg6-2*. Scale bar = 5 cm.** D** Plant height. **E** Tiller number. **F** Primary branch number per panicle. **G** Secondary branch number per panicle. **H** Grain number per panicle. **I** 1000-grain weight. From **D** to **I**, values are means ± SD (*n* = 15). *P < 0.05; **P < 0.01; ns, no significant difference (one-way analysis of variance (ANOVA))

There were no significant differences in shoot length (Fig. 3E) or root length (Fig. 3F) among *osrglg6-1*, *osrglg6-2*, and ZH11. To determine whether *OsRGLG6* was involved in the regulation of rice grain number and yield, we examined the agronomic traits of *osrglg6-2* and ZH11. *osrglg6-2* showed reductions in panicle size, grain number, and leaf angle compared with ZH11, but it did not differ significantly from ZH11 in plant height. Statistical analyses revealed that *osrglg6-2* had significantly more tillers but fewer secondary branches and grains per panicle than ZH11 and showed no significant differences in plant height, primary branch number, or thousand-grain weight (Fig. 4A–I). These results suggest that *OsRGLG6* specifically controls grain number in rice.

### OsRGLG6 controls rice grain number by promoting OsOTUB1 degradation

**Fig. 5:**
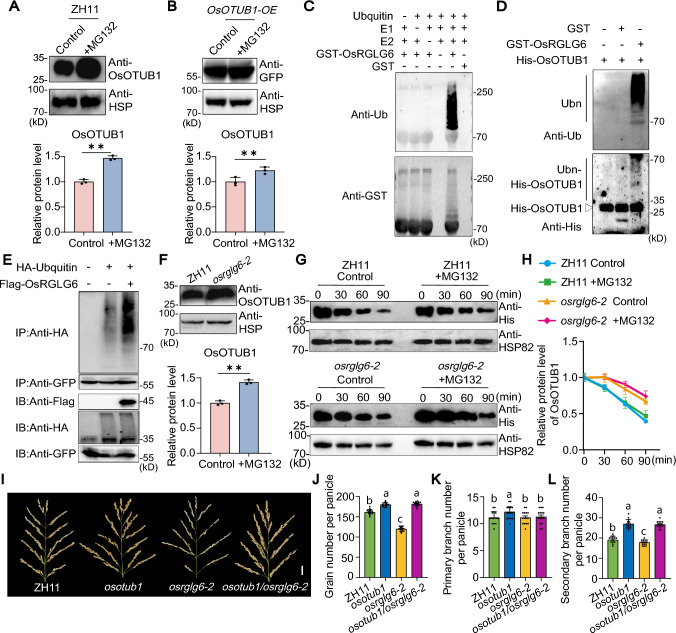
OsRGLG6 mediates the ubiquitination and degradation of OsOTUB1. **A** and **B** The proteasome inhibitor MG132 (50 μM) stabilizes OsOTUB1 protein in ZH11 **A** and *OsOTUB1-OE* **B** seedlings. HSP82 served as a loading control.** C** In vitro ubiquitination assay demonstrating the E3 ligase activity of GST-OsRGLG6 in the presence of ubiquitin, E1, and E2 (UBcH5b). GST-OsRGLG6 was detected with both anti-ubiquitin and anti-GST antibodies. **D** In vitro ubiquitination of His-OsOTUB1 by GST-OsRGLG6. His-OsOTUB1 and GST-OsRGLG6 were detected using anti-His and anti-GST antibodies, respectively. **E** In vivo ubiquitination assay in protoplasts from *pActin::OsOTUB1-GFP* transgenic rice plants, showing increased OsOTUB1 ubiquitination by OsRGLG6. **F** OsOTUB1 protein levels were higher in *osrglg6-2* than in ZH11. Total protein was extracted from 2-week-old ZH11 and *osrglg6-2* seedlings*.* **G** In vitro degradation experiment with OsOTUB1. Cell lysates of ZH11 and *osrglg6-2* were incubated with recombinant His-OsOTUB1 fusion protein. HSP82 was used as a loading control. **H** Quantitative analysis of the changes in OsOTUB1 content shown in **G** (*n* = 3). **I** Panicle morphology of ZH11, *osotub1*, *osrglg6-2*, and *osotub1/osrglg6-2*. Scale bar = 2 cm. **J** Grain number per panicle. **K** Primary branch number per panicle. **L** Secondary branch number per panicle. In **A**, **B** and **F**, values are means ± SD (*n* = 3), *P < 0.05; **P < 0.01(one-way analysis of variance (ANOVA). In **J**–**L**, values are means ± SD (*n* = 15), different lowercase letters denote significant differences determined by Tukey’s HSD (*P* < 0.05)

Previous research has demonstrated the presence of ubiquitination on OsOTUB1 (Zhu et al. 2020). To determine whether OsOTUB1 is degraded via the 26S proteasome pathway, we measured changes in OsOTUB1 protein content after addition of the proteasome inhibitor MG132 to seedlings of ZH11 and OsOTUB1-overexpression lines (*OsOTUB1-OE*). OsOTUB1 protein levels increased significantly following MG132 treatment, confirming that OsOTUB1 is degraded via the ubiquitin 26S proteasome pathway (Fig. 5A, B).

We next performed in vitro ubiquitination experiments to confirm the E3 ligase activity of the OsRGLG6 protein. Immunoblotting with anti-ubiquitin antibodies revealed the presence of ubiquitinated proteins only when all reaction components (ubiquitin, E1, E2, and GST-OsRGLG6) were included. Furthermore, immunoblotting with anti-GST antibody revealed additional higher-molecular-weight protein bands in the presence of E1, E2, and GST-OsRGLG6 (Fig. 5C). These findings indicate that OsRGLG6 can undergo auto-ubiquitination in the presence of E1 and E2 enzymes, thereby confirming its function as an E3 ubiquitin ligase.

To determine whether OsRGLG6 can ubiquitinate OsOTUB1, we performed in vitro and in vivo ubiquitination experiments. In the presence of E1 and E2, OsRGLG6 added free ubiquitin molecules to OsOTUB1 to form polyubiquitinated OsOTUB1, demonstrating that OsRGLG6 ubiquitinates OsOTUB1 in vitro (Fig. 5D). Subsequent in vivo ubiquitination experiments showed that the addition of Flag-OsRGLG6 increased the ubiquitination level of OsOTUB1 in protoplasts from *OsOTUB1-GFP* transgenic seedlings, indicating that OsRGLG6 promotes the ubiquitination of OsOTUB1 in vivo (Fig. 5E). These findings imply that OsRGLG6 may promote the degradation of OsOTUB1 through the 26S proteasome pathway. To identify the E2 ubiquitin-conjugating enzymes involved in OsRGLG6-mediated ubiquitination of OsOTUB1, we tested four E2 candidates (OsUBC13, OsUBC15, OsUBC16, and OsUBC22) for interaction with OsRGLG6. Y2H and BiFC assays confirmed that OsRGLG6 interacted with all four E2 enzymes in both the cytoplasm and endomembrane system (Fig. S4A, B).

We next analyzed the protein and transcript levels of OsOTUB1 in *osrglg6-2* and ZH11 (Fig. 5F, Fig. S5). *OsOTUB1* transcript levels were comparable in *osrglg6-2* and ZH11, but OsOTUB1 protein levels were significantly higher in *osrglg6-2*, suggesting that OsRGLG6 promotes OsOTUB1 degradation. To confirm these results, we performed in vitro degradation-tracking experiments and found that His-OsOTUB1 was more stable in total protein extracts of *osrglg6-2* than in those of ZH11, and MG132 treatment slowed OsOTUB1 degradation (Fig. 5G, H). Collectively, these results support the conclusion that OsRGLG6 promotes the ubiquitination and degradation of OsOTUB1. To test the genetic relationship between *OsRGLG6* and *OsOTUB1* in the control of rice grain number and panicle branching, we examined the panicle phenotypes of the *osrglg6-2/osotub1* double mutant, *osrglg6-2*, *osotub1*, and ZH11. Grain number and secondary branch number of the *osrglg6-2/osotub1* double mutant were similar to those of *osotub1* and were significantly higher than those of ZH11 and *osrglg6-2*, whereas primary branch number of double mutant resembled those of *osrglg6-2* (Fig. 5I–L). These results suggest that OsRGLG6 regulates grain number in rice by promoting the ubiquitination and degradation of OsOTUB1.

### The *OsRGLG6*–*OsOTUB1* module may influence phytohormone signaling, nitrogen metabolism, and abiotic stress responses

**Fig. 6:**
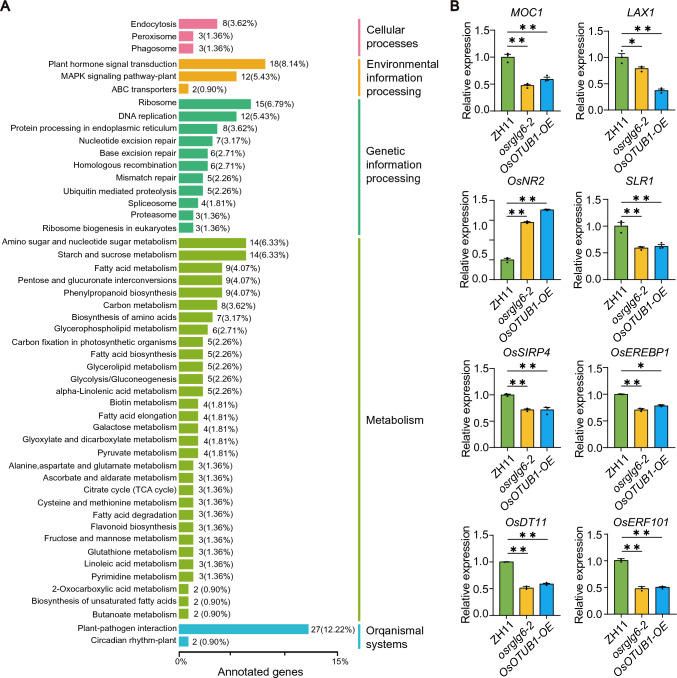
Transcriptional regulation by the *OsRGLG6–OsOTUB1* module. **A** KEGG pathway annotations of genes differentially expressed between ZH11 and *osrglg6-2*. Total RNA was extracted from 2-week-old ZH11 and *osrglg6-2* seedlings; two independent biological replicates were included for each genotype*.* **B** Relative expression levels of *MOC1*, *LAX1*, *OsNR2*, *SLR1*, *OsSIRP4*, *OsEREBP1*, *OsDT11*, and *OsERF101* in ZH11, *osrglg6-2*, and *OsOTUB1-OE*. Total RNA was extracted from 2-week-old ZH11, *osrglg6-2*, and *OsOTUB1-OE* seedlings, and three independent biological replicates were analyzed for each genotype. Values are means ± SEM (*n* = 3). *P < 0.05; **P < 0.01 (one-way analysis of variance (ANOVA))

To investigate the downstream target genes regulated by OsRGLG6, we performed transcriptome analysis, which identified 806 genes whose expression differed significantly between *osrglg6-2* and ZH11 (119 upregulated and 687 downregulated). We assigned GO terms and KEGG pathways to these differentially expressed genes and found that many were associated with the KEGG pathways plant hormone signal transduction (ko04075), ribosome (ko03010), amino sugar and nucleotide sugar metabolism (ko00520), starch and sucrose metabolism (ko00500), and plant*–*pathogen interaction (ko04626) (Fig. 6A, Fig. S6, Fig. S7).

To identify downstream target genes co-regulated by *OsRGLG6* and *OsOTUB1-OE*, we screened for potential candidates based on their expression patterns in *osrglg6-2* and *OsOTUB1-OE* plants, their functions, and examined their expression by RT–qPCR (Fig. 6B). Notable candidates included *MOC1* and *LAX1*, which regulate grain number and tiller number (Zhang et al. 2021; Wang and Li 2008; Komatsu et al. 2003); *OsNR2*, which is associated with nitrogen uptake and utilization (Gao et al. 2019); *SLR1*, a negative regulator of gibberellin (GA) signaling (Itoh et al. 2002); and stress-responsive genes such as *OsSIRP4* (Kim et al. 2021), *OsEREBP1* (Jisha et al. 2015), *OsDT11* (Li et al. 2017), and *OsERF101* (Jin et al. 2018).

The expression of *MOC1* and *LAX1* was significantly downregulated in *osrglg6-2* and *OsOTUB1-OE* plants, suggesting that *OsRGLG6* and *OsOTUB1* might co-regulate branch number and grain number through these genes. By contrast, *OsNR2* expression was significantly upregulated in *osrglg6-2* and *OsOTUB1-OE* plants, indicating that *OsRGLG6*, *OsOTUB1*, and *OsNR2* might jointly regulate nitrogen uptake and utilization in rice and that *OsRGLG6* might enhance these processes. In addition, *SLR1* expression was significantly reduced in *osrglg6-2* and *OsOTUB1-OE*, indicating that *OsRGLG6* might act as a negative regulator of GA signal transduction. The expression levels of *OsSIRP4*, *OsEREBP1*, *OsDT11*, and *OsERF101* were also significantly lower in *osrglg6* and *OsOTUB1-OE* plants, suggesting that *OsRGLG6* might positively regulate responses to abiotic stresses such as drought and salt stress.

### *OsRGLG6 *and *OsOTUB1* co-ordinately regulate drought responses

**Fig. 7:**
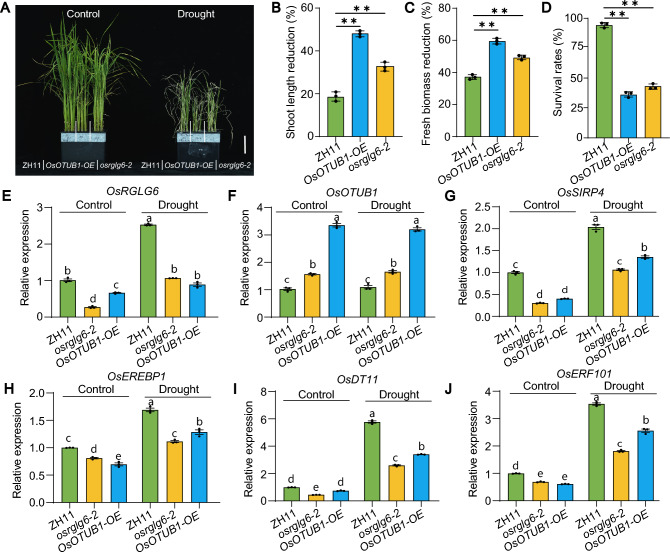
Drought sensitivity and stress-gene regulation in *osrglg6-2* and *OsOTUB1-OE* seedlings. **A** Phenotypes of ZH11, *osrglg6-2*, and *OsOTUB1-OE* after simulated drought treatment. Scale bar = 5 cm. **B–D** Percentage reductions in shoot length **B**, percentage reductions in biomass **C**, and survival rates **D** of ZH11, *osrglg6-2*, and *OsOTUB1-OE* under drought stress. **E–J** Relative expression levels of *OsRGLG6*, *OsOTUB1*, *OsSIRP4*, *OsDT11*, *OsERF101*, and *OsEREBP1* in ZH11, *osrglg6-2*, and *OsOTUB1-OE* under control and drought conditions. Total RNA was extracted from 2-week-old control and drought-treated seedlings of ZH11, *osrglg6-2*, and *OsOTUB1-OE*. In **B**, **C** and **D**, values are means ± SD (*n* = 3), **P < 0.01 (one-way analysis of variance (ANOVA). From **E** to **J**, values are means ± SEM (*n* = 3), different lowercase letters denote significant differences determined by Tukey’s HSD (*P* < 0.05)

To investigate whether *OsRGLG6* and *OsOTUB1* are involved in the drought-stress response, we exposed *osrglg6-2*, *OsOTUB1-OE*, and ZH11 seedlings to simulated drought conditions. Under drought stress, *osrglg6-2* and *OsOTUB1-OE* showed greater percentage reductions in plant height and fresh biomass than ZH11, and their survival rates were significantly lower, demonstrating that they were sensitive to drought stress (Fig. 7A–D, Fig. S8). To examine whether *OsRGLG6* and *OsOTUB1* transcript levels were drought responsive, we quantified the expression of both genes in ZH11, *osrglg6-2*, and *OsOTUB1-OE* seedlings under drought stress (Fig. 7E, F). Drought stress increased the transcript levels of *OsRGLG6* but had no effect on those of *OsOTUB1*, suggesting that OsRGLG6 responds to drought at the transcriptional level, whereas OsOTUB1 responds at the protein level. To determine whether *OsRGLG6* and *OsOTUB1* mediate drought-stress responses by regulating *OsSIRP4*, *OsEREBP1*, *OsDT11*, and *OsERF101*, we analyzed the expression of these genes in ZH11, *osrglg6-2*, and *OsOTUB1-OE* under drought. The drought-induced upregulation of *OsSIRP4*, *OsEREBP1*, *OsDT11*, and *OsERF101* observed in ZH11 was compromised in both *osrglg6-2* and *OsOTUB1-OE* seedlings, suggesting that *OsRGLG6* and *OsOTUB1* function together to mediate the drought-responsive transcription of these downstream genes (Fig. 7G–J). To determine whether the drought-stress response regulated by *OsRGLG6* and *OsOTUB1* is dependent on the ABA signaling pathway, we transiently treated ZH11 seedlings with ABA and analyzed the expression levels of multiple ABA-related genes: *OsRGLG6*, *OsOTUB1*, *OsPP2C06* (Li et al. 2015), *OsPYL4* (Tian et al. 2015), *OsSIRP4*, *OsEREBP1*, *OsDT11*, and *OsERF101*. Expression of *OsRGLG6*, *OsPP2C06*, *OsPYL4*, *OsSIRP4*, *OsEREBP1*, *OsDT11*, and *OsERF101* was induced by ABA, although expression of *OsOTUB1* was not, indicating that the drought-stress response regulated by *OsRGLG6* and *OsOTUB1* is dependent on the ABA signaling pathway (Fig. S9). These results indicate that OsRGLG6 and OsOTUB1 co-ordinately regulate drought responses by modulating the transcription of genes involved in drought and ABA signaling pathways. Collectively, these results suggest that *OsRGLG6* plays a pivotal role in various physiological processes in rice, including growth regulation and environmental stress adaptation..

## Discussion

The number of grains per panicle is a critical determinant of rice yield, and enhancing this trait remains a key objective for breeders (Li et al. 2022, 2023). However, the molecular mechanisms that regulate grain number in rice are not fully understood. Here, we screened for proteins that interacted with OsOTUB1, a key regulator of grain number in rice, and identified the E3 ubiquitin ligase OsRGLG6. OsRGLG6-GFP was predominantly localized to the cytoplasm and ER and was highly expressed in young panicles. Knockout of *OsRGLG6* reduced grain number and yield, suggesting that increased *OsRGLG6* expression could potentially boost rice productivity.

Many earlier studies of grain-number regulation focused on genes related to hormone signaling pathways (e.g., auxin and cytokinin signaling); such genes mainly regulate grain number by influencing panicle branching and floral organ development (Ashikari et al. 2005; Kurakawa et al. 2007; Sandhu et al. 2024; Guo et al. 2020a). By contrast, OsRGLG6 acts through the ubiquitination pathway, interacting with OsOTUB1 and facilitating its ubiquitination-mediated degradation. OsOTUB1, a deubiquitinating enzyme, participates in the regulation of multiple agronomic traits, including tiller number, grain number per panicle, and grain weight (Huang et al. 2017; Wang et al. 2017). Downregulation of *OsOTUB1* can increase the number of grains per panicle and boost rice yield. Mechanistically, OsOTUB1 interacts with the transcription factor OsSPL14 and limits its K63-linked ubiquitination, which in turn promotes its K48-linked ubiquitination and proteasomal degradation (Wang et al. 2017). Overexpression of *OsSPL14* promotes rice panicle branching and increases yield (Miura et al. 2010), and OsSPL14 transcriptionally regulates *DEP1*, a key gene controlling grain number per panicle in rice (Jiao et al. 2010; Lu et al. 2013). These results suggest that *OsRGLG6*, *OsOTUB1*, *OsSPL14*, and *DEP1* may form a molecular network that regulates grain number and yield in rice. This network also provides a potential strategy for enhancing crop yield by manipulating the expression of *OsRGLG6* and its homologs in other crops.

*OsRGLG6* and *OsOTUB1* co-regulate multiple downstream target genes with known roles in grain-number determination, nitrogen uptake and utilization, gibberellin signaling, and responses to biotic and abiotic stresses. This implies that *OsRGLG6* not only modulates grain number but also has far-reaching effects on other biological processes. Among the downstream genes examined, those encoding the salt-responsive E3 ubiquitin ligase OsSIRP4, the drought-responsive AP2/ERF transcription factors OsEREBP1 and OsERF101, and the cysteine-rich peptide OsDT11 were all downregulated in *osrglg6-2* and *OsOTUB1-OE* lines. Overexpression of *OsEREBP1*, *OsDT11*, and *OsERF101* enhances drought tolerance in rice (Jisha et al. 2015; Li et al. 2017; Jin et al. 2018), whereas overexpression of *OsSIRP4* reduces rice salt tolerance (Kim et al. 2021). It is, therefore, likely that OsRGLG6 and OsOTUB1 influence salt- and drought-stress responses by regulating the transcription of these genes. Transient ABA treatment induced the expression of *OsRGLG6*, *OsSIRP4*, *OsEREBP1*, *OsDT11*, and *OsERF101*, suggesting that *OsRGLG6* participates in the ABA-mediated regulation of downstream genes and that *OsRGLG6* shows functional conservation with its Arabidopsis homologs *AtRGLG1* and *AtRGLG5* (Wu et al. 2016a, b). PEG-mediated drought-simulation experiments demonstrated that OsRGLG6 likely modulates the drought response by regulating OsOTUB1 protein stability in rice. Collectively, these results indicate that the OsRGLG6–OsOTUB1 module plays a critical role in coordinating drought stress responses and panicle development in rice and that this regulatory mechanism is evolutionarily conserved in diverse species. Because OsRGLG6 co-ordinately enhances both yield (through GPP regulation) and drought tolerance (Fig. 7), it could potentially be used to overcome the typical trade-off between yield and stress resistance, making it a promising target for precision breeding.

Our Y2H and BiFC assays also revealed that OsRGLG6 interacts with OsUBC13, an E2 ubiquitin-conjugating enzyme known to regulate grain number in rice. Overexpression of *OsUBC13* increases grain number, and its reduced expression has the opposite effect (Wang et al. 2017). Given that OsUBC13 also interacts with OsRGLG6, we propose that OsOTUB1, OsUBC13, and OsRGLG6 may co-ordinately regulate grain number in rice. This coordinated regulation underscores the complexity of the ubiquitination pathway in rice and highlights the importance of understanding the interplay between E3 ligases, deubiquitinating enzymes, and their substrates.

In conclusion, our results demonstrate that OsRGLG6 ubiquitinates OsOTUB1 to promote its degradation, thereby regulating grain number in rice. This study not only reveals a previously unknown mechanism for the control of grain number but also provides genetic resources for the development of high-yielding rice varieties. Future research should focus on exploring the broader interaction network involving OsRGLG6 and other components of the ubiquitination pathway, as well as investigating its regulatory mechanisms under different environmental conditions. This work will contribute to improving rice productivity and enhancing stress resilience in agricultural settings.

## Materials and methods

### Plant materials and growing conditions

Paddy-grown plants were spaced 20 cm apart and grown during the standard growing season at two experimental stations, one in Lingshui (Hainan Province) and one in Hefei (Anhui Province). Rice seedlings were grown in a hydroponic culture system using rice nutrient solution (NH_4_Cl, 1.5 mmol/L; Ca(NO_3_)_2_·4H_2_O, 0.75 mmol/L; NaH_2_PO_4,_ 0.5 mmol/L; K_2_SO_4,_ 0.76 mmol/L; CaCl_2,_ 1 mmol/L; MgSO_4_·7H_2_O, 1.67 mmol/L; Fe-EDTA(Na), 0.04 mmol/L; H_3_BO_3,_ 19 μmol/L; (NH_4_)_6_Mo_7_O_24_·4H_2_O, 0.52 μmol/L; MnSO_4_·H_2_O, 9.1 μmol/L; CuSO_4_·5H_2_O, 0.16 μmol/L), and the culture medium was replaced every 3 days.

### Transgene constructs

Gene-specific spacer sequences (20 bp) derived from *OsRGLG6* were inserted into the *pYL-U6a-gRNA* entry vector and subcloned into the destination vector *pYLCRISPR/Cas9-MT* with Cas9 by simultaneous enzymatic cutting and ligation. All transgenic rice plants were created by *Agrobacterium*-mediated transformation (Wang et al. 2017). The *OsOTUB1-OE* overexpression line was constructed as previously described (Wang et al. 2017). The relevant primer sequences are provided in Table S2.

### Genotyping of *osrglg6* mutants

Mutant candidates were genotyped by PCR using the gene-specific primers cecrOsRGLG6F and cecrOsRGLG6R, and two homozygous mutants, *osrglg6-1* and *osrglg6-2*, were identified (Table S2).

### Bioinformatics analysis of RGLG protein

Protein domains in OsRGLG6 were predicted using the InterPro website (https://www.ebi.ac.uk/interpro/). The amino acid sequences of RGLGs from rice were obtained from the NCBI database (https://www.ncbi.nlm.nih.gov), and those of RGLGs from Arabidopsis were retrieved from TAIR (https://www.arabidopsis.org/index.jsp). A phylogenetic tree was constructed with the neighbor-joining method and 1000 bootstrap replicates using TBtools (Chen et al. 2020). Conserved amino acid motifs in *OsRGLG1–6* were identified using the MEME website (http://meme-suite.org/tools/meme) and visualized using TBtools.

### RT-qPCR

Total RNA was extracted from 2-week-old plant tissues using the TRIzol reagent (Invitrogen) and treated with RNase-free DNase I (Invitrogen) according to the manufacturer’s protocol. The resulting RNA was reverse transcribed using a cDNA synthesis kit (TransGen Biotech, AE311). RT-qPCR was performed using SYBR Green Real-Time PCR Master Mix (TransGen Biotech, AQ601) with three independent RNA preparations as biological replicates. Real-time PCR data were obtained using the CFX96 Touch Real-Time PCR Detection System (Bio-Rad) and analyzed using the 2^−ΔΔCt^ method. The cycling parameters were 2 min at 95 °C, followed by 40 amplification cycles of 5 s at 95 °C and 30 s at 60 °C. Rice *Actin1* was used as the reference gene. The relevant primer sequences are provided in Table S2.

### Y2H assay

The Y2H assay was performed using the Matchmaker Gold Yeast Two-Hybrid System (Takara Bio USA) following the manufacturer’s instructions. The full-length *OsOTUB1.1* coding sequence (CDS) and a 597-bp C-terminal fragment were amplified from ZH11 cDNA and inserted into *pGBKT7* (Takara), and the full-length CDSs of *OsRGLG6*, *OsUBC13*, and the other three *E2* gene*s* were inserted into *pGADT7* (Takara). Each of these plasmids were confirmed by sequencing, then transformed into yeast strain AH109. The required β-galactosidase assays were performed according to the manufacturer’s protocol. Cells harboring an empty *pGBKT7* or empty *pGADT7* vector were used as the negative controls. The full-length *OsOTUB1* sequence or the C-terminal fragment were used as bait to screen a cDNA library prepared from poly(A) RNA isolated from young rice panicles (< 0.2 cm in length). Screening and plasmid isolation were performed according to the manufacturer’s protocol. The relevant primer sequences are provided in Table S2.

### Subcellular localization assay

The *OsRGLG6* CDS without the stop codon was inserted into the *p35S::GFP-1300* vector by the Gateway LRR reaction (primer details are provided in Table S2). Nine-day-old rice seedlings were digested with 20 mL of enzyme mixture (1.5% [w/v] cellulase R-10, 0.5% [w/v] macerozyme R-10, 5 mM 2-morpholinoethanesulfonic acid, 0.1% [w/v] BSA, 10 mM CaCl_2_, and 0.6 M mannitol, pH 5.8) and incubated at 28 °C for 5 h in the dark. Purified rice protoplasts were then transfected with *p35S::OsRGLG-GFP-1300* by PEG-mediated transformation. Subcellular localization assays were also performed by agroinfiltration of *N. benthamiana* epidermal cells as described previously (Wang et al. 2017). Fluorescent signals were observed using an ultra-high-speed confocal cell imaging system (Dragonfly CR-DFLY-505).

### BiFC assay

Versions of *OsOTUB1.1*, *OsRGLG6*, *OsUBC13*, and three other *E2s* with and without stop codons were amplified from cDNA of ZH11, and the amplicons were inserted into the *pCAMBIA2300-35S-cYFP-nos* or *pCAMBIA2300-35S-nYFP-nos* vector using the Gateway system to generate a set of fusion constructs. The plasmids were introduced into *Agrobacterium tumefaciens* strain GV3101 for transient transformation into *N. benthamiana.* The YFP signal was examined and photographed under a confocal microscope (Andor Dragonfly). Each BiFC assay was repeated at least three times. The relevant primer sequences are listed in Table S2.

### In vitro pull-down assay

The full-length CDS of *OsRGLG6* was fused to the N terminus of GST in the *pGEX4T-1* vector, and the full-length CDS of *OsOTUB1* was fused to the C terminus of His in the *pET-28a* vector. These plasmids were transformed into *Escherichia coli* strain BL21, which was cultured in LB liquid medium with shaking until the OD_600_ reached 0.5. IPTG was then added to induce recombinant protein expression at 28 °C for 8 h. The bacterial cells were collected by centrifugation. After addition of extraction buffer (PBS for GST-OsRGLG6 and NTA-0 for His-OsOTUB1), the cells were disrupted using an ultrasonic homogenizer (Sonics,VCX800). The supernatant was obtained by centrifugation. The GST-OsRGLG6 fusion protein was purified by binding to glutathione Sepharose beads and eluted with a reduced glutathione solution. His-OsOTUB1 was purified by binding to Ni–NTA agarose beads and eluted with different gradient concentrations of imidazole solution. The recombinant GST-OsRGLG6 fusion protein was immobilized on glutathione Sepharose beads and incubated with His-OsOTUB1 for 60 min at 4 °C. The beads were washed three times, and the proteins were eluted with elution buffer (50 mM Tris–HCl, 10 mM reduced glutathione, pH 8.0). The supernatant was immunoblotted using anti-His (Diluted 1:2000, Santa Cruz, sc-8036) and anti-GST antibodies (Diluted 1:2000, Santa Cruz, sc-138).

### Co-immunoprecipitation and western blotting

The full-length CDS of *OsRGLG6* was fused to the *pCAMBIA1300-35S-GFP-nos* vector, and the full-length CDS of *OsOTUB1* was fused to the *pCAMBIA2300-Actin-Flag-nos* vector. These plasmids were subsequently introduced into *A. tumefaciens* strain GV3101 for transformation into *N. benthamiana*. Following a 36-h dark incubation period, proteins were extracted using a buffer consisting of 50 mM HEPES (pH 7.5), 150 mM KCl, 1 mM EDTA, 0.5% Triton-X 100, 1 mM DTT, and protease inhibitors (Roche Life Science, Basel, Switzerland) for 30 min. After incubation with agarose-conjugated anti-GFP antibody (Sigma-Aldrich) at 4 °C for at least 4 h, the mixture was washed five or six times with TBS-T buffer and eluted with 2 × protein loading buffer (TransGen Biotech, DL101-02). The immunoprecipitate and input fraction were subjected to SDS-PAGE, and the separated proteins were transferred to nitrocellulose membranes (GE Healthcare). The GFP-OsRGL6 fusion protein was detected with anti-GFP antibody (Diluted 1:5000, Roche, 11,814,460,001), and the Flag-OsOTUB1 fusion protein was detected with anti-Flag antibody (Diluted 1:5000, Sigma-Aldrich, F1804).

### Analysis of OsOTUB1 degradation

Lysates were obtained from 2-week-old ZH11 and *osrglg6* seedlings using a lysis buffer consisting of 25 mM Tris HCl (pH 7.5), 10 mM NaCl, 10 mM MgCl_2_, 4 mM PMSF, 5 mM DTT, and 10 mM ATP, and then incubated with the appropriate recombinant His-OsOTUB1 fusion protein. The mixtures were exposed to 50 µM MG132 for predetermined times or left untreated, then subjected to SDS-PAGE and western blotting using an anti-His antibody (Santa Cruz, sc-8036). HSP82 was detected with an anti-HSP82 antibody (BGI) as a loading control.

### Analysis of in vitro ubiquitination

Reaction mixtures containing E1 (50 ng,E-305–025, R&D), E2 (500 ng, E2-622–100, R&D), 2 μg/μL ubiquitin (U-100H-10 M, R&D), and purified GST-OsRGLG6 (500 ng) were mixed in 1 × reaction buffer (50 mM Tris HCl (pH 7.4), 10 mM MgCl_2_, 5 mM ATP, and 2 mM DTT). The reactions were incubated at 28 °C for 0.5 h, and in vitro E3 ligase activity was determined using anti-Ub (Diluted 1:3000, Abcam, ab134953) and anti-GST (Diluted 1:5000, Santa Cruz, sc-138) antibodies. For the substrate ubiquitination assay, equal amounts of purified His-OsOTUB1 were added to the reaction mixture, and ubiquitination was measured using anti-Ub (Diluted 1:3000, Abcam, ab134953) and anti-His (Diluted 1:2000, Santa Cruz, sc-8036) antibodies.

### Analysis of in vivo ubiquitination

Rice protoplasts prepared from *pActin::OsOTUB1-GFP* transgenic seedlings were transfected with *pUC19-35S-HA-Ubiq-RBS* in the presence or absence of the *pUC19-35S-Flag-OsRGLG6* plasmid. After 15 h, the protoplasts were lysed in extraction buffer (50 mM Tris–HCl (pH 7.40), 150 mM KCl, 1 mM EDTA, 0.5% Triton-X 100, and 1 mM DTT) with proteinase inhibitor cocktail (Roche Life Science). The resulting lysates were incubated with GFP-Nanoab-Agarose Beads (Novozymes) for at least 4 h at 4 °C, rinsed five or six times in extraction buffer, and eluted by boiling in 2 × protein loading buffer (TransGen Biotech, DL101-02). The immunoprecipitates were separated by SDS-PAGE and transferred to a nitrocellulose membrane (GE Healthcare) for western blotting with anti-GFP (Diluted 1:5000, Roche, 11,814,460,001), anti-HA (Diluted 1:2000, Santa Cruz, sc-7392), and anti-Flag conjugate (Diluted 1:5000, Sigma-Aldrich, F1804) antibodies.

### DEGs and functional enrichment analyses

ZH11 and *osrglg6-2* plants were cultured in rice nutrient solution for 14 days. Shoots from five plants at a similar developmental stage were pooled to create one replicate. This process was performed three times for each genotype, resulting in a total of six samples (three replicates per genotype). After extraction of total RNA from each sample, sequencing libraries were constructed using standard RNA-seq protocols, and RNA sequencing was performed by Beijing BioMarker Technologies. The Clean Reads obtained from sequencing are aligned with the reference genome (Oryza_sativa. IRGSP_1. 0_RAPDB_2024. 1. genome. fa) using the HISAT2 software to acquire the positioning information of the Reads on the reference genome. Transcript abundance was quantified as fragments per kilobase of exon model per million mapped reads (FPKM) using the StringTie software, and transcripts with FPKM ≥ 1 were considered to be expressed. Differentially expressed genes (DEGs) were identified using the DESeq2 R package (Love et al. 2014). To control for multiple testing, the false discovery rate (FDR) was set to 0.001, and genes with an FDR-adjusted *P* value of < 0.001 and a |log_2_(fold change)|≥ 1 were classified as differentially expressed (Ren et al. 2024). GO enrichment and KEGG pathway annotation were performed on differentially expressed genes using the ClusterProfiler software. KEGG pathway analysis was performed, retaining pathways containing ≥ 2 genes; GO enrichment was filtered by a *P* value cutoff of 0.01.

### Drought stress and ABA treatment

After germination, ZH11, *osrglg6-2*, and *OsOTUB1-OE* seedlings were grown in rice nutrient solution for 2 weeks and then the experimental group was subjected to simulated drought stress using 20% PEG6000, while the control group continued cultivation in standard nutrient solution. After 10 days, plant height, biomass, and survival rate were recorded for each line. Two-week-old ZH11 seedlings were treated with 10 μM abscisic acid (ABA) dissolved in nutrient solution. Whole seedlings were harvested at specified time intervals (3 h and 6 h post-treatment), immediately frozen in liquid nitrogen, and stored at −80 °C for subsequent RNA extraction.

### Data analysis

All data were analyzed by one-way analysis of variance (ANOVA) or Tukey’s HSD performed in Microsoft Excel 2019 (Microsoft, Redmond, WA, USA).

### Accession numbers

Sequence data from this article can be found in the MSU Rice Genome Annotation Project Database under the following accession numbers: *OsRGLG6*, *LOC_Os08g38600*; *OsOTUB1*, *LOC_Os08g42540*; *MOC1*, *LOC_Os06g40780*; *LAX1*, *LOC_Os01g61480*; *OsNR2*, *LOC_Os02g53130*; *SLR1*, *LOC_Os03g49990*; *OsSIRP4*, *LOC_Os04g01490*; *OsEREBP1*, *LOC_Os02g54160*; *OsDT11*, *LOC_Os11g10590*; *OsERF101*, *LOC_Os04g32620*; *OsPP2C06, LOC_Os01g40094*; *OsPYL4, LOC_Os03g18600*.

## Supplementary Information

Below is the link to the electronic supplementary material.

Supplementary file1 (DOCX 3755 KB)

Supplementary file2 (XLSX 11 KB)

Supplementary file3 (XLSX 10 KB)

## Data Availability

All data that support the conclusions of this research are contained within the article and its supplementary materials.
