# Supplementary material for: The RING-domain E3 ubiquitin ligase OsRGLG6 regulates rice grain number and yield via ubiquitination-mediated degradation of OsOTUB1
Source: aBIOTECH. 2025 Jul 23;6(4):774–89. doi: 10.1007/s42994-025-00232-5 (PMC12647413; doi:10.1007/s42994-025-00232-5)
Supplement: Supplementary file 1 — Supplementary file1 (DOCX 3755 KB) [file 42994_2025_232_MOESM1_ESM.docx]

**The RING-domain E3 ubiquitin ligase OsRGLG6 regulates rice grain number and yield via ubiquitination-mediated degradation of OsOTUB1**

**Jia Chen^1^, Huixia Song^1^, Chenyang Xu^1^, Pengfei Wang^2^,** **Shuansuo Wang^1^**

^1^Shanxi Houji Laboratory, Shanxi Agricultural University, Taiyuan 030031, China

^2^Journal Center, Shanxi Agricultural University, Taiyuan 030031, China

Corresponding author: Shuansuo Wang, sswang@sxau.edu.cn

Co-corresponding author: Pengfei Wang, [wangpengfei39@sxau.edu.cn](mailto:wangpengfei39@sxau.edu.cn)

**This file includes:**

1, Legend of Supplementary Tables

2, Supplementary Figure 1 to 9

**Supplemental Table S1 Putative OsOTUB1 interactors identified in a yeast two-hybrid library screen**

**Supplemental Table S2 Primers used in this study.**

**Supplementary figure**


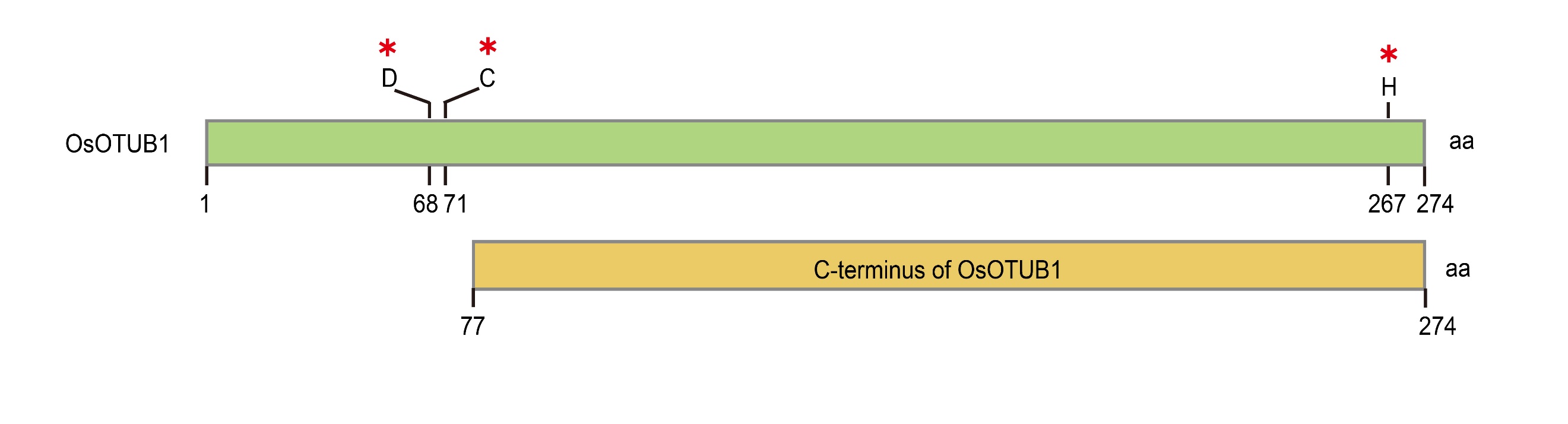


**Fig. S1** Schematic diagram of OsOTUB1. N-terminal deletion of OsOTUB1 disrupts its enzymatic activity. The red asterisks indicate the putative catalytic triad of OsOTUB1.


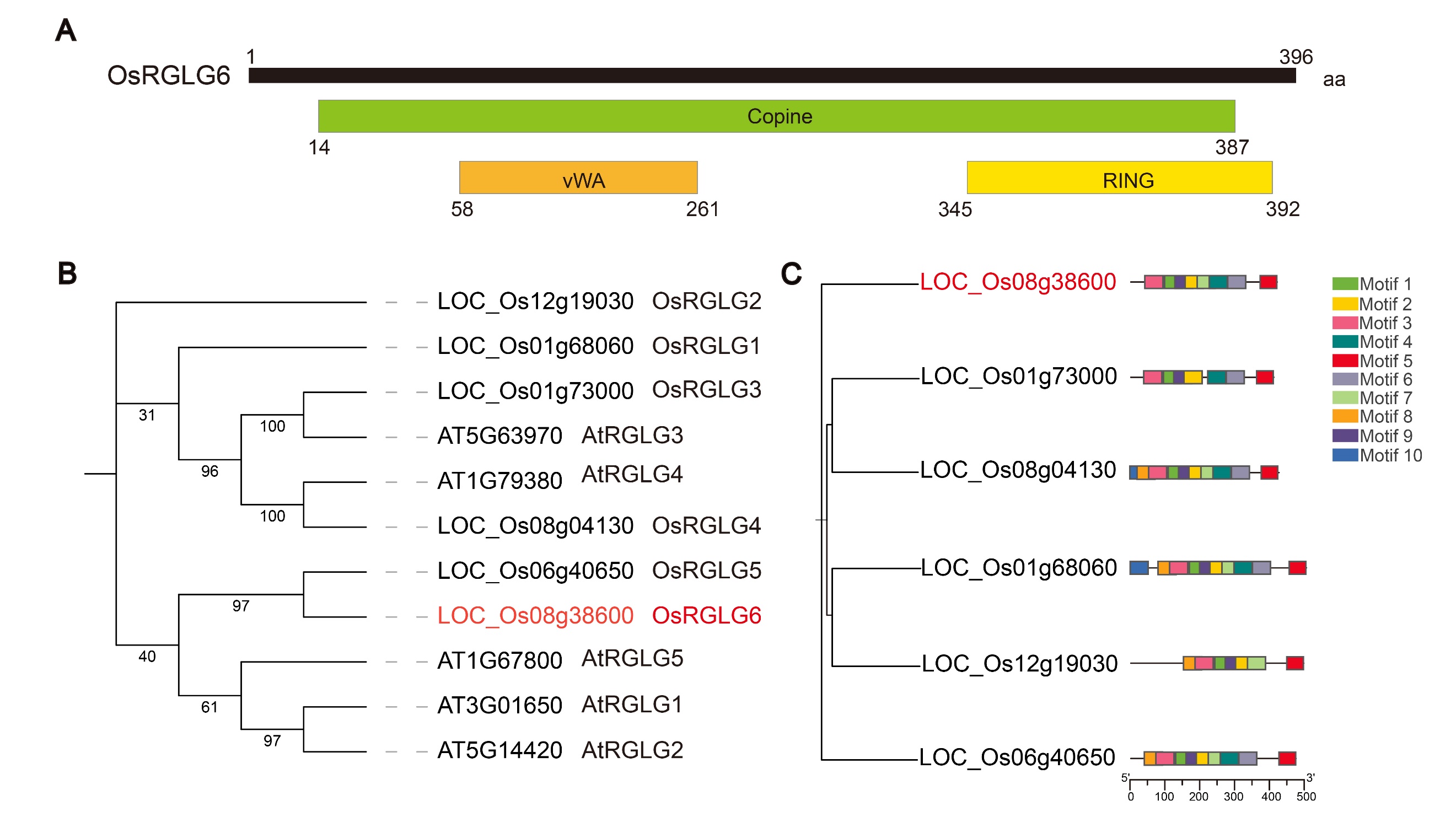


**Fig. S2** OsRGLG6 is a RING-type E3 ubiquitin ligase. **A** Schematic diagram of the OsRGLG6 protein. OsRGLG6 contains a vWA domain and a RING domain. **B** Phylogenetic tree of RGLG proteins from Arabidopsis and rice generated by the neighbor-joining method with 1000 bootstrap replicates. **C** Ten conserved motifs identified in the rice OsRGLG proteins.


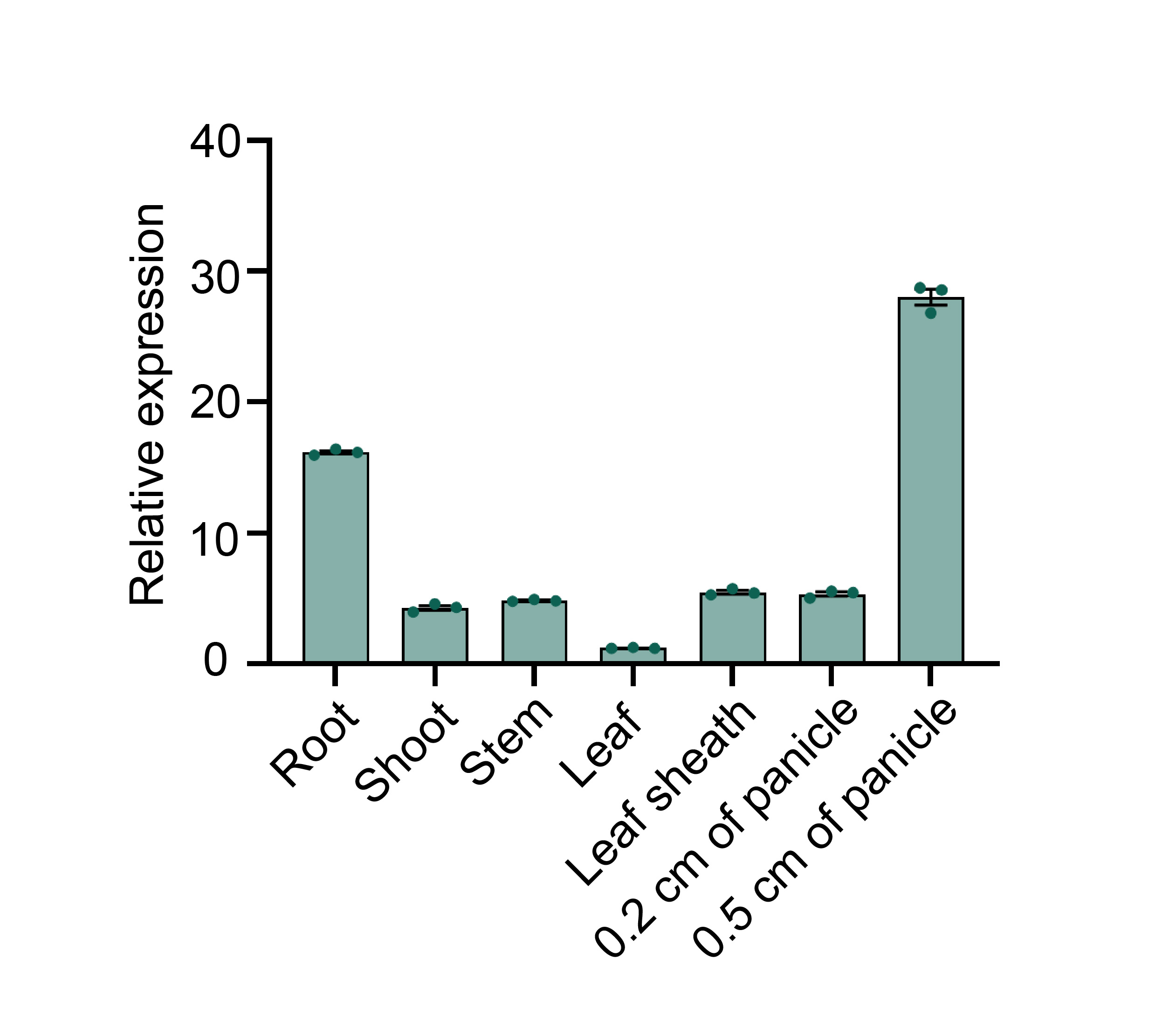


**Fig. S3** Relative expression levels of *OsRGLG6* in roots, shoots, stems, leaves, leaf sheaths, and panicles at two developmental stages (0.2 and 0.5 cm). Values are means ± SEM (*n* = 3).


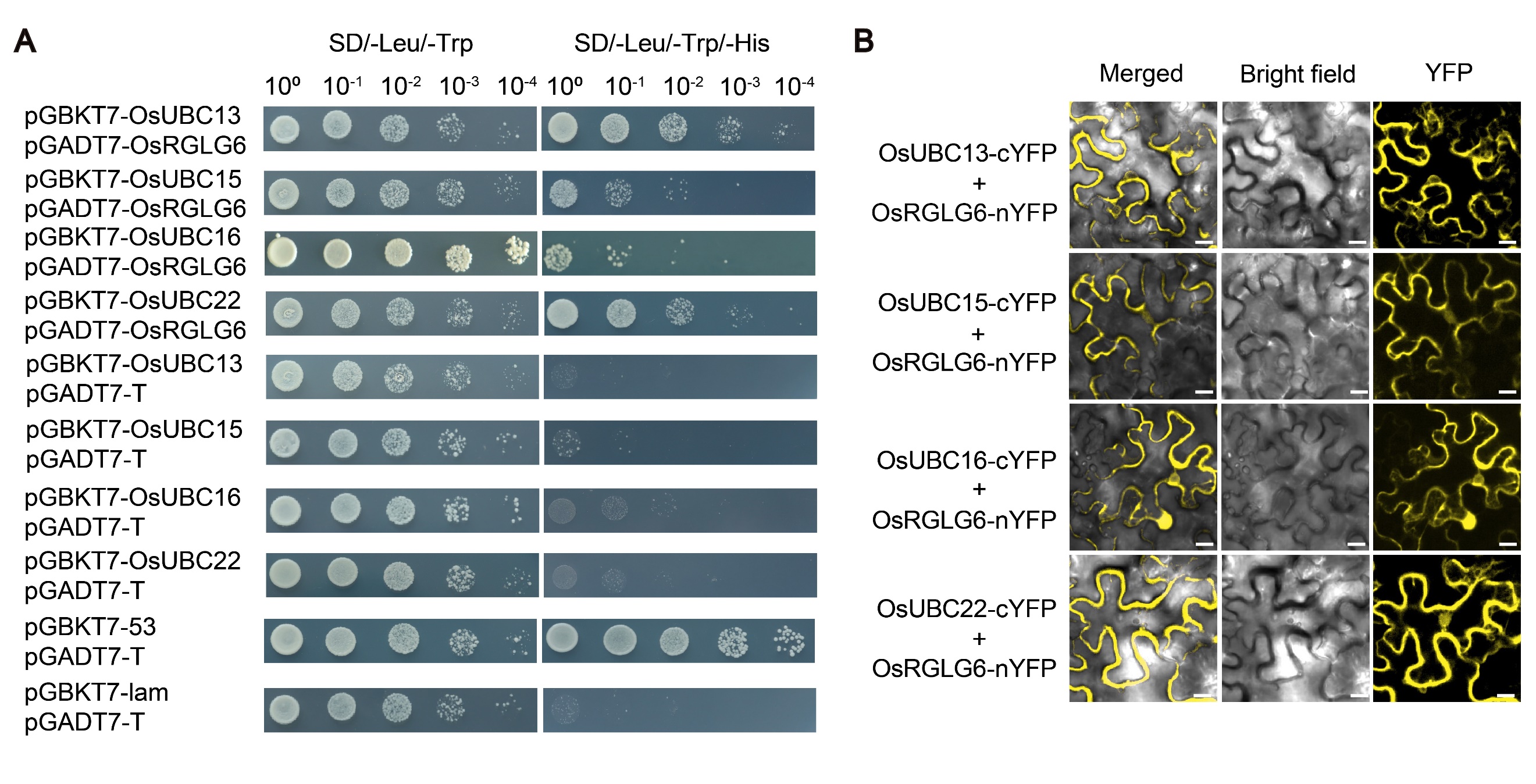


**Fig. S4** OsRGLG6 interacts with E2 ubiquitin-binding enzymes. **A** Yeast two-hybrid assay showing interactions of OsRGLG6 with OsUBC13, OsUBC15, OsUBC16, and OsUBC22. The yeast cells were grown on SD/–Leu/–Trp (left) and SD/–Leu/–Trp/–His media (right). **B** BiFC assay showing the interactions of OsRGLG6 with OsUBC13, OsUBC15, OsUBC16, and OsUBC22 in *N. benthamiana*. Each BiFC assay was performed at least three times (scale bars = 25 μm).


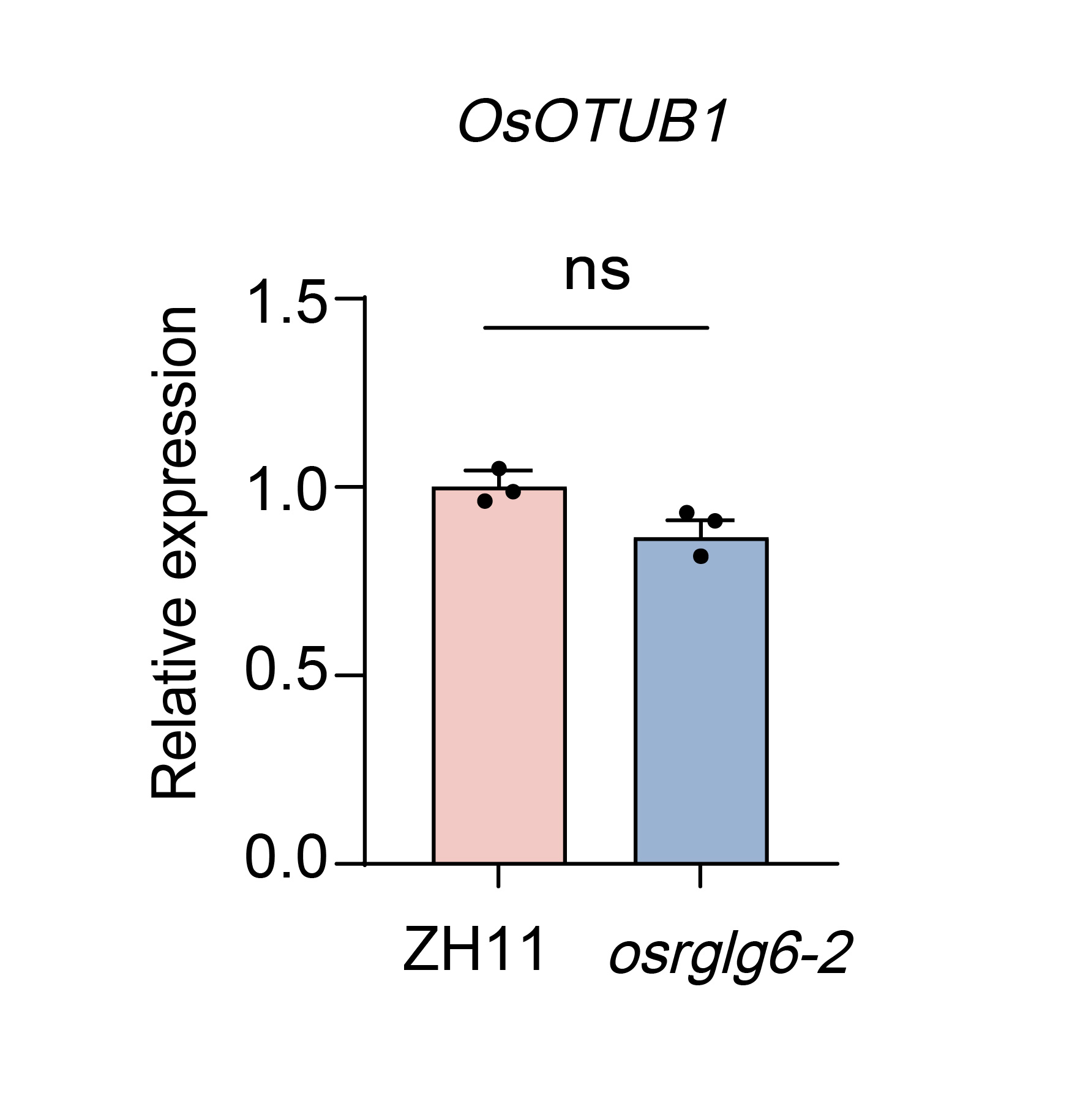


**Fig. S5** Relative expression levels of *OsOTUB1* in ZH11 and *osrglg6-2*. Values are means ± SEM (*n* = 3). ns, no significant difference (one-way analysis of variance (ANOVA)).


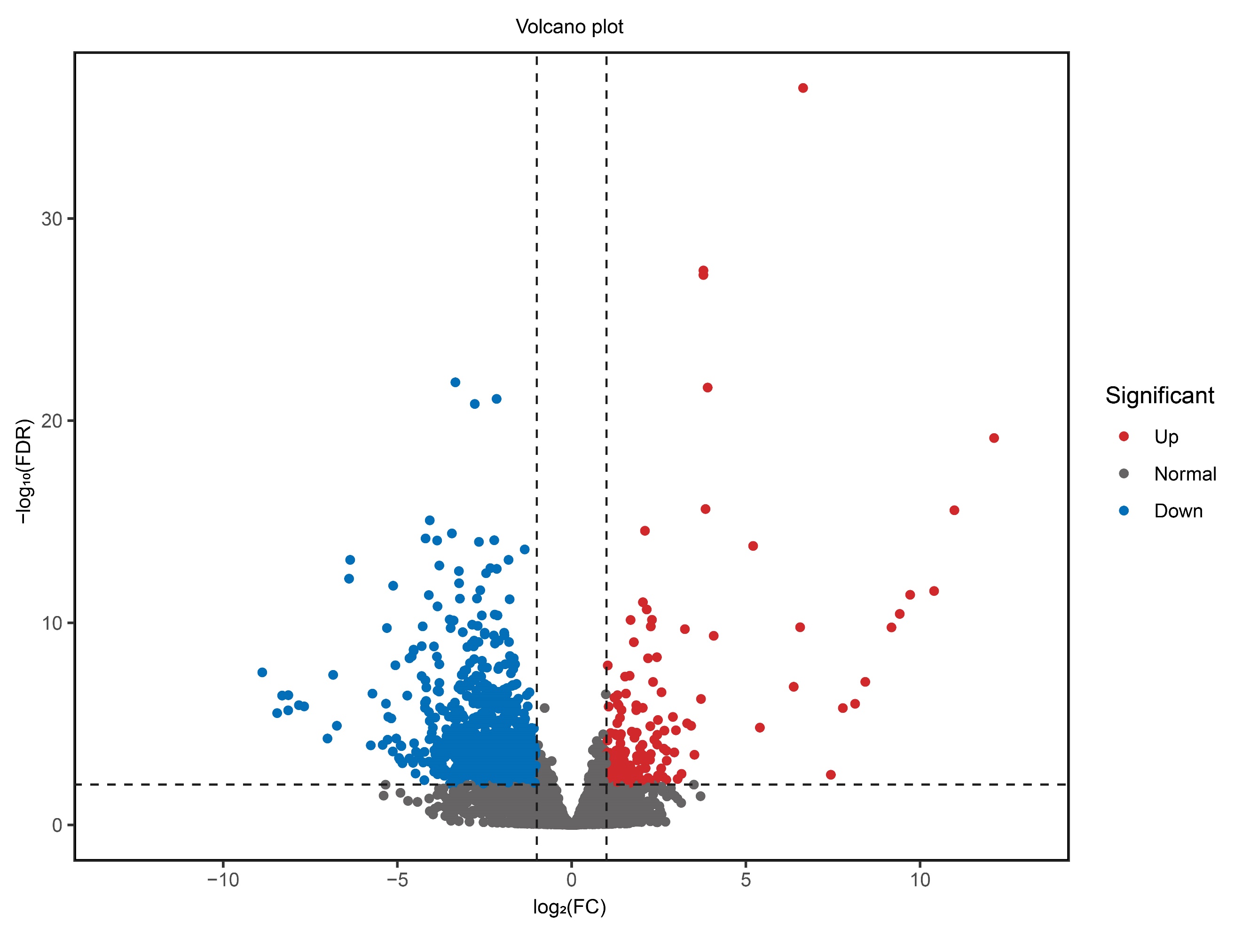


**Fig. S6** Volcano plot showing differential gene expression between ZH11 and *osrglg6-2*.


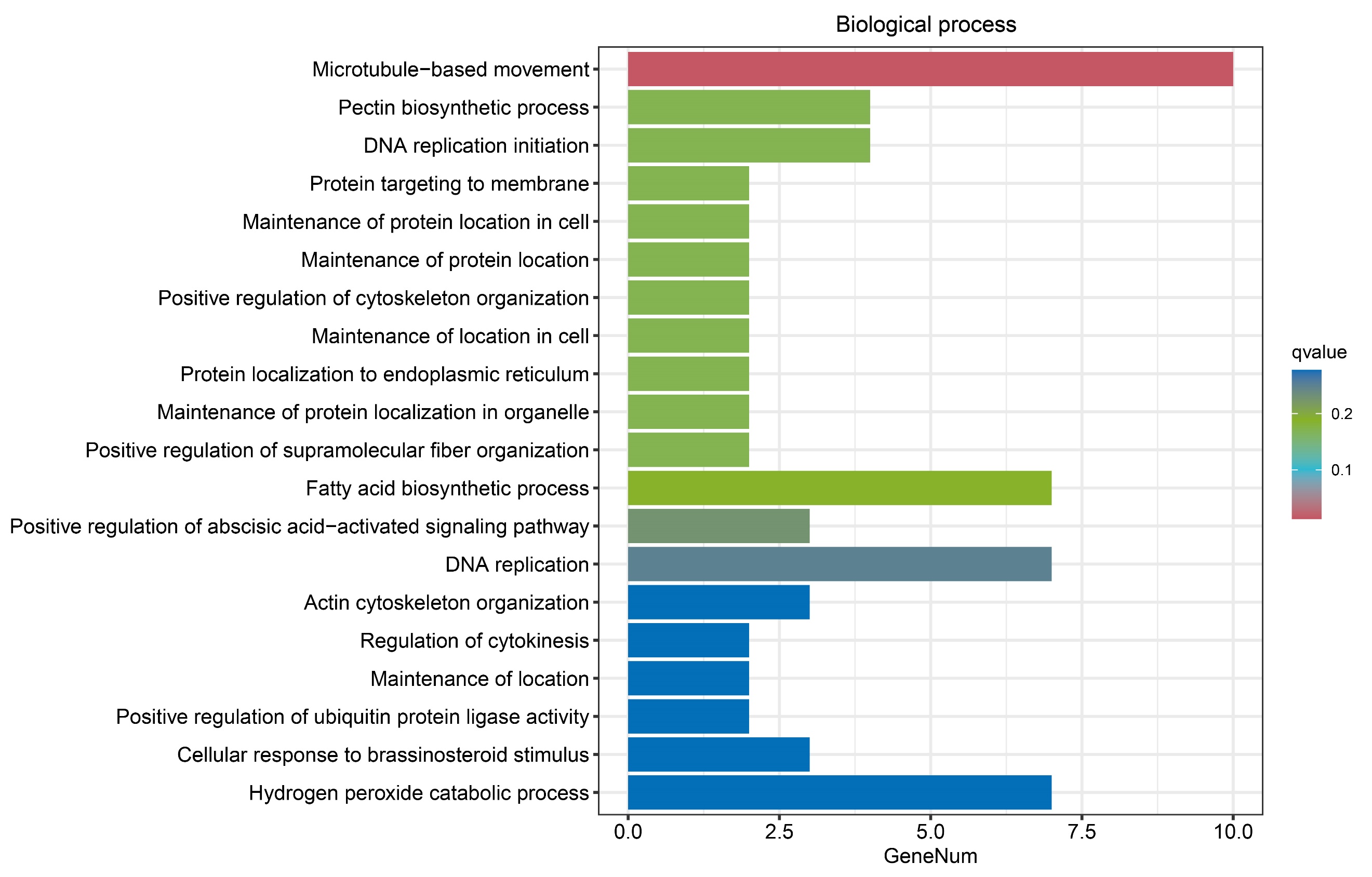


**Fig. S7** GO enrichment analysis of genes differentially expressed between ZH11 and *osrglg6-2*.


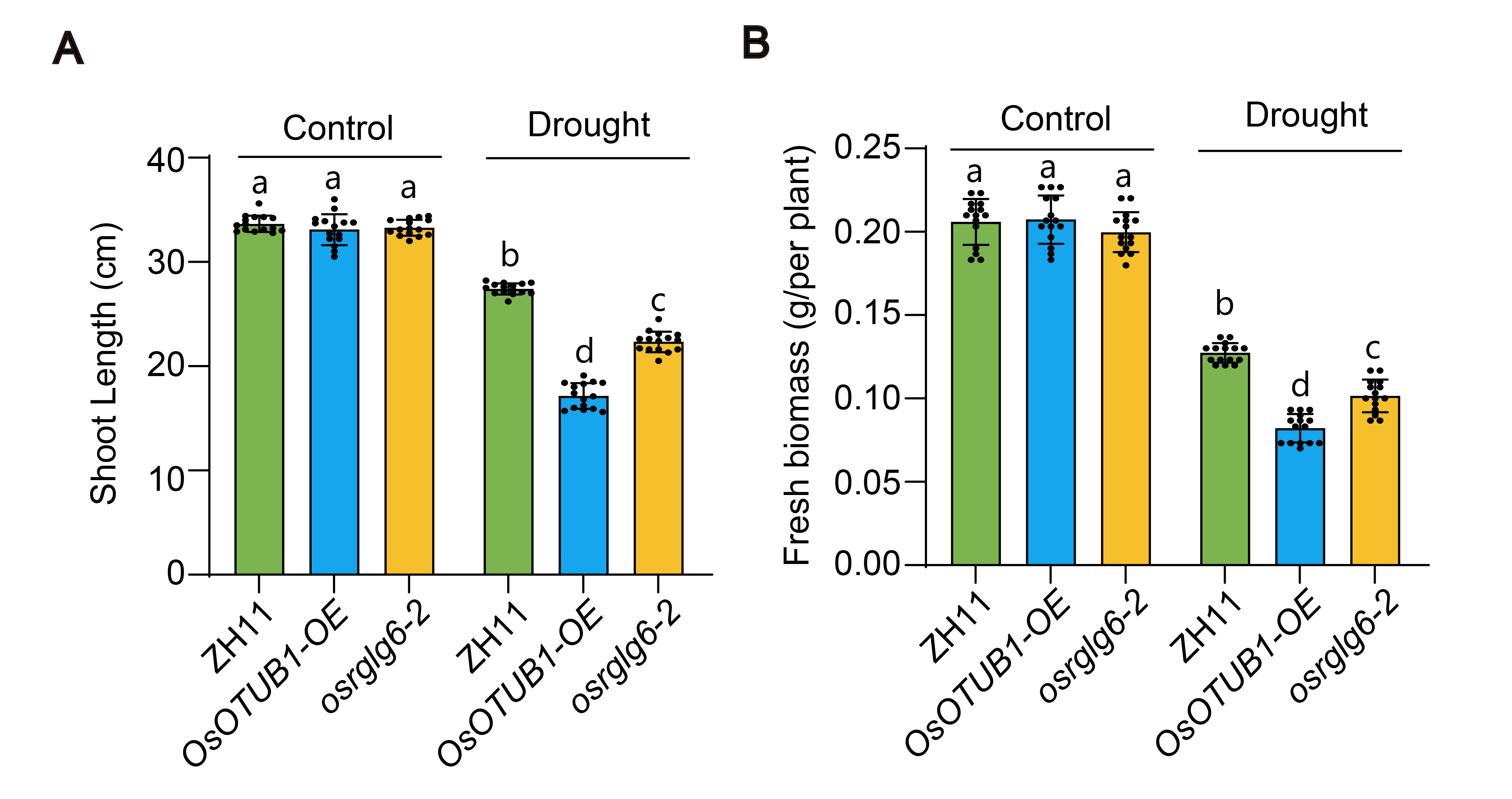


**Fig. S8** Phenotypes of ZH11, *osrglg6-2*, and *OsOTUB1-OE* after simulated drought treatment. **A, B** Shoot length (**A**) and fresh biomass (**B**) of ZH11, *OsOTUB1-*OE, and *osrglg6-2* under control and drought conditions. Values are means ± SEM (*n* = 15). different lowercase letters denote significant differences determined by Tukey’s HSD (*P*<0.05).


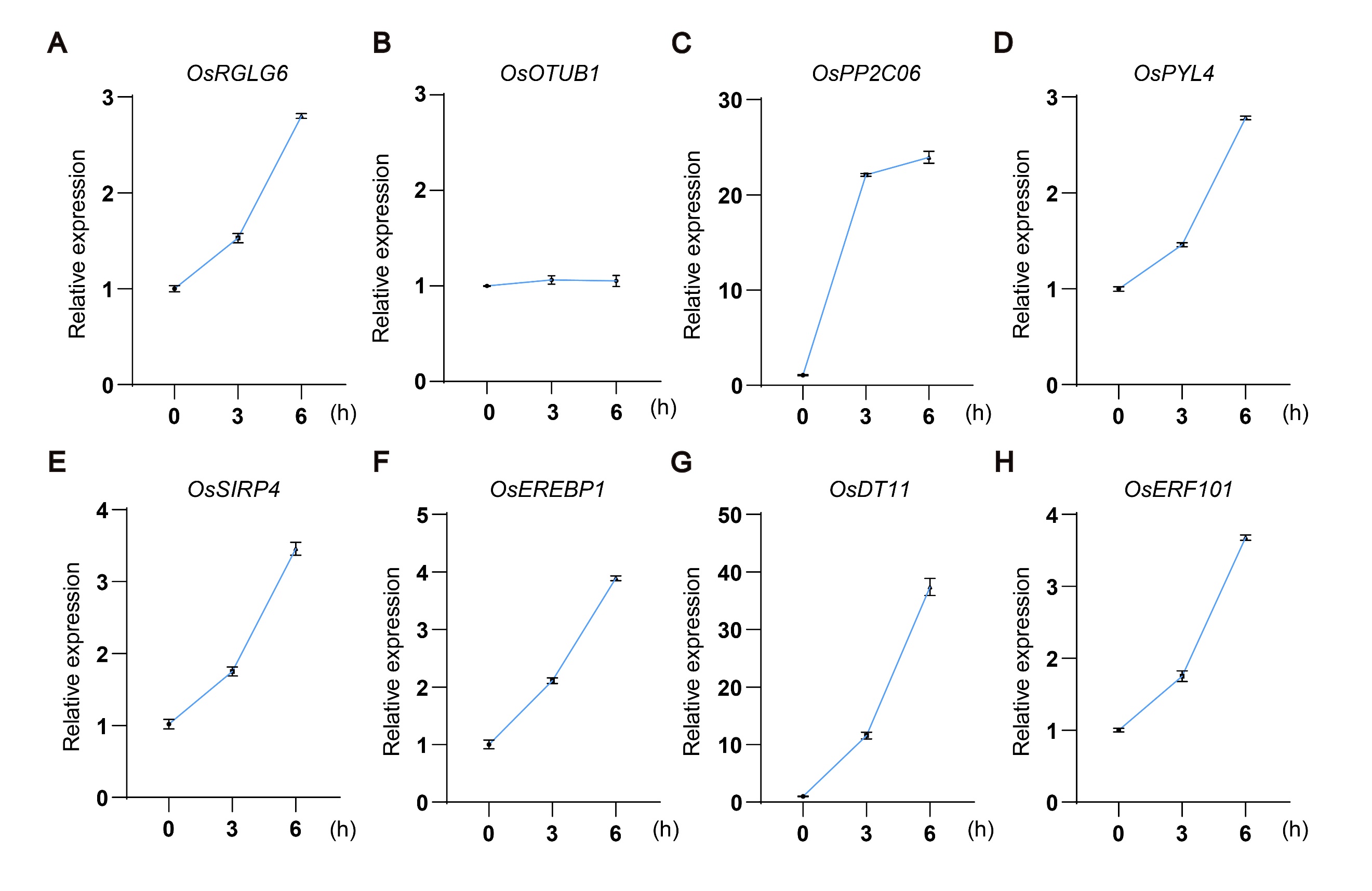


**Fig. S9** Relative expression levels of *OsRGLG6*, *OsOTUB1*, *OsPP2C06*, *OsPYL4*, *OsSIRP4*, *OsEREBP1*, *OsDT11*, and *OsERF101* in ZH11, *osrglg6-2*, and *OsOTUB1-OE* after ABA treatment. Values are means ± SEM (*n* = 3).
